# Combined analysis of the transcriptome and metabolome provides insights into the fleshy stem expansion mechanism in stem lettuce

**DOI:** 10.3389/fpls.2022.1101199

**Published:** 2022-12-15

**Authors:** Ying Huang, Yanwen Li, Zhenning Liu, Wanqin Chen, Yalin Wang, Xiaohua Wang, Yihua Liu, Yangxia Zheng

**Affiliations:** ^1^ College of Agriculture and Forestry Sciences, Linyi University, Linyi, China; ^2^ College of Horticulture, Sichuan Agricultural University, Chengdu, China; ^3^ College of Hydraulic and Environmental Engineering, China Three Gorges University, Yichang, China

**Keywords:** stem lettuce, fleshy stem expansion, anatomical analysis, metabolic regulation, gene expression

## Abstract

As a stem variety of lettuce, the fleshy stem is the main product organ of stem lettuce. The molecular mechanism of fleshy stem expansion in stem lettuce is a complex biological process. In the study, the material accumulation, gene expression, and morphogenesis during fleshy stem expansion process were analyzed by the comparative analysis of metabolome, transcriptome and the anatomical studies. The anatomical studies showed that the occurrence and activity of vascular cambium mainly led to the development of fleshy stems; and the volume of pith cells gradually increased and arranged tightly during the expansion process. A total of 822 differential metabolites and 9,383 differentially expressed genes (DEGs) were identified by the metabolomics and transcriptomics analyses, respectively. These changes significantly enriched in sugar synthesis, glycolysis, and plant hormone anabolism. The expression profiles of genes in the sugar metabolic pathway gradually increased in fleshy stem expansion process. But the sucrose content was the highest in the early stage of fleshy stem expansion, other sugars such as fructose and glucose content increased during fleshy stem expansion process. Plant hormones, including IAA, GA, CTK, and JA, depicted important roles at different stem expansion stages. A total of 1,805 DEGs were identified as transcription factors, such as MYB, bHLH, and bZIP, indicating that these transcription factor families might regulate the fleshy stems expansion in lettuce. In addition, the expression patterns identified by qRT-PCR were consistent with the expression abundance identified by the transcriptome data. The important genes and metabolites identified in the lettuce fleshy stem expansion process will provide important information for the further molecular mechanism study of lettuce fleshy stem growth and development.

Lettuce, an annual or biennial herb belonging to the *Lettuce* genus of the Compositae family, is widely cultivated throughout China. As an important vegetable and medicinal plant, lettuce is rich in vitamin C, anthocyanins, and flavonoids (Malarz et al., 2020; Medina-Lozano et al., 2021). Lettuce can be classified as a leaf or stem lettuce according to its edible organs. The stem diameter of stem lettuce is an important commercial trait and directly affects its quality and economic value. Therefore, it is necessary to explore the molecular mechanism of stem expansion process.

The formation and development of plant stems is a complex physiological process which can be regulated by nutrients, hormones, genes, and environmental factors. The type and ratio of sugar and acid also affects the quality and commercial value of stem lettuce. Soluble sugar and organic acids are important nutrients and flavor substances of fruits and vegetables, which also participate in the process of plant metabolism (Das, 2015; Das et al., 2019; Ahmed et al., 2020). During the fruit ripening period of ‘Orin’ apples, the content of four sugars (glucose, sucrose, fructose, and sorbitol) and the malic acid increased (Yang et al., 2021). As the important signaling substances, plant hormones could regulate plant development, physiology, growth, and reproduction (Durbak et al., 2012; Wu et al., 2022). Numerous studies have demonstrated that hormones such as auxin (IAA), gibberellin (GA), abscisic acid (ABA), cytokinin (CTK), and methyl jasmonate (MeJA) participated in the process of rhizome development (Saidi and Hajibarat, 2021; Chen et al., 2022). For example, CTK promoted the formation and development of potato tubers and significantly increased the tuberization rate (Pavlista, 2011). JA and MeJA promoted the expansion of apical cells and tubers by inhibiting stolon elongation (Sohn et al., 2011). In the process of carrot root development, GA-related genes were differentially expressed, which indicated that GA might play a crucial role in carrot elongation and expansion (Wang et al., 2015).

A variety of metabolites and genes have been confirmed to participate in the expansion process of plant organs (root, stem). During mustard stem development process, most differentially expressed genes (DEGs) were identified to involve in the synthesis, accumulation, and metabolism process of sugars, starch, and storage proteins (Li et al., 2020). A large number of genes have been identified to involve in the process of rhizome development. For instance, radish *CycD3* and *RsCLE22a* gene were confirmed to participate in the formation of fleshy roots (Lutova et al., 2008; Dong et al., 2022). Two *MADS-box* genes could regulate vascular cambium activity and secondary growth of poplar by regulating IAA homeostasis (Zheng et al., 2020). The initiation and activity of *Arabidopsis thaliana* cambium were controlled by the transcriptional regulator AHL15 (Rahimi et al., 2022). Tomato *SD1* gene played positively roles in tomato stem development by regulating the size and number of secondary phloem cells (Ye et al., 2020). The transcription factor (TF) gene *SlHB8* negatively regulated tomato stem development by inhibiting xylem width and xylem cell layers (Liu et al., 2021).

Previous studies on stem lettuce have mainly focused on the effect of light on the lettuce quality (Li et al., 2021; Qi et al., 2021). The molecular mechanism of fleshy stem expansion in stem lettuce is unclear. In the study, the anatomical structure changes during fleshy stem expansion of stem lettuce were analyzed. The combined analysis of metabolomo and transcriptome were also conducted to determine changes in the metabolites and genes at four fleshy stem expansion stages (S1: transverse diameter length of fleshy stem is 1 cm, S2: transverse diameter length of fleshy stem is 2 cm, S3: transverse diameter length of fleshy stem is 3 cm, S4: transverse diameter length of fleshy stem is 4 cm). The results will provide a theoretical basis for elucidating the expansion mechanism of fleshy stems in stem lettuce.

## Materials and methods

### Plant materials and treatment

In this study, the cultivated stem lettuce ‘Yonganhong’ was selected as the experimental material. The seeds were placed in the incubator for 12 h photoperiod at 22℃ and 18℃ (day vs. night) with a light intensity of 20,000 µmol/m^2^/s (lux) to raise seedlings. When the seedlings grew to 2-3 true leaves, the healthy seedlings with the same growth were selected and transplanted into the nutrition bowl (23 cm × 18 cm). Then, the seedlings were transferred into the greenhouse to grow normally under natural light. The fleshy stem of stem lettuce began to expand as time goes on. To ensure the accuracy of experimental data, digital calipers were chosen to measure the fleshy stem diameter of the third internode. When the diameter of the swollen stem at this internode reached to 1cm (S1 stage), 2cm (S2 stage), 3cm (S3 stage), and 4cm (S4 stage), the samples were collected and frozen in liquid nitrogen for further analysis.

### Anatomical structure of fleshy stem in stem lettuce

The fleshy stems of the stem lettuce ‘Yonganhong’ at four expansion stages (S1, S2, S3, and S4) were fixed in 50 mL 50% FAA fixative and placed at 4°C for 24 h. Then, the treated samples were embedded in paraffin and stained with safranin O-fast green reagent referring to previous methods (Li et al., 2020). The anatomical structure during the process of fleshy stem expansion was observed and recorded with CaseViewer (version 7.3).

### Determination of plant hormones

Plant hormones, including IAA, ABA, JA, CTK, and GA, were extracted at four expansion stages (S1, S2, S3, and S4) of fleshy stem in stem lettuce. Each expansion stage contained three biological replicates. The endogenous hormones were quantified by Nanjing Ruiyuan Biotechnology Co., Ltd according to the previously described method (Liu et al., 2010).

### Metabolite profiling and data analysis

The extraction of metabolites and metabolomics analysis was conducted by Biomarker Technologies Co., Ltd (Beijing, China). Six biological replicates at each expansion stages of fleshy stem (S1, S2, S3, and S4) were collected for the metabolomics analysis. The samples after liquid nitrogen grinding were added 500 μL extraction solution (methanol: acetonitrile = 1:1), followed by ultrasonic for 10 min and incubation at -20℃ for 1 h, then centrifuging to obtain the supernatants. The supernatants were dried in a vacuum concentrator, then added 160 μL extraction solution (acetonitrile: water = 1:1) for further analysis. Samples of equal volume were taken from each experimental sample and mixed to obtain quality control samples (QC), inserted before, during and after the samples to test the repeatability of the experiment. The liquid chromatography-mass spectrometry system in this study was composed of ultra-high performance liquid chromatography (Waters Acquity I-Class PLUS) and high resolution mass spectrometer (Waters Xevo G2-XS QTOF). The chromatographic and mass spectrometric conditions were as follows: Acquity UPLC HSS T3 column (1.8 µm, 2.1×100 mm, Waters) was used, the injection volume was 1 µl, and the flow rate was 400 μL/min. Mobile phase A was 0.1% formic acid aqueous solution, mobile phase B was 0.1% formic acid acetonitrile. The chromatographic gradient elution procedure consisted of 0~0.25 min, 98% A, 2% B; 0.25~10.00 min, 2% A, 98% B; 10.00~13.00 min, 2% A, 98% B; 13.00~13.10 min, 98% A, 2% B; 13.10~15.00 min, 98% A, 2% B. The mass spectrometer collected primary and secondary mass spectrum data in MSe mode controlled by acquisition software (MassLynx version 4.2, Waters). The data was analyzed by principal component analysis (PCA), partial least squares discrimination analysis (PLS-DA), and other multivariate statistical analysis. Then, the variable importance in the projection (VIP), fold change (FC), and p-value were used to screen metabolites using the following screening criteria: FC > 1.0, p < 0.05, and VIP > 1.0. HMDB (https://hmdb.ca/metabolites) and LIPIDMAPS database were used to conduct the annotation of the functions and classifications of the metabolites. The bioinformatics analysis of the metabolites was conducted using BMKCloud (www.biocloud.net).

### Transcriptome data analysis

A total of 12 samples of four fleshy stem expansion stages (S1, S2, S3, and S4) with three biological replicates were subjected to RNA sequencing. The Agilent 2100 bioanalyzer was used to accurately detect the integrity and total amount of RNA. The cDNA libraries (S1, S2, S3, and S4) were sequenced on the Illumina HiSeq 4000 sequencing platform (Biomarker Technologies Co., Ltd., Beijing, China), as previously described (Zhang et al., 2017). FPKM (fragments per kilobase of transcript per million fragments mapped) was used as an index to measure the level of transcripts or gene expression. DESeq2 (version 1.20.0) was used to analyze the differential expression between sample groups. FC represented the ratio of the expression between two samples (stages). The false discovery rate (FDR) was obtained by correcting the p-value of the significance of the difference, and the screening criteria were FC ≥ 2 and FDR < 0.01. The bioinformatics analysis of the DEGs was conducted using BMKCloud (www.biocloud.net).

### qRT-PCR analysis

The total RNA was extracted from the four fleshy stem expansion stages (S1, S2, S3, and S4) and subjected to qRT-PCR analysis using the Roche LightCycler 96 and the SYBR qPCR master mix. The amplification procedure was as follows: pre-denaturation at 95°C for 30 s, then 40 cycles of denaturation at 95°C for 10 s, and annealing at 60°C for 30 s. Gene expression was normalized using *LsTIP41* as an internal reference and quantified using 2^−ΔΔCt^ as described previously (Pfaffl, 2001; Borowski et al., 2014). The primers used for qRT-PCR were listed in **Table S1**.

## Results

### Anatomical analysis of lettuce fleshy stem

**Figure 1:**
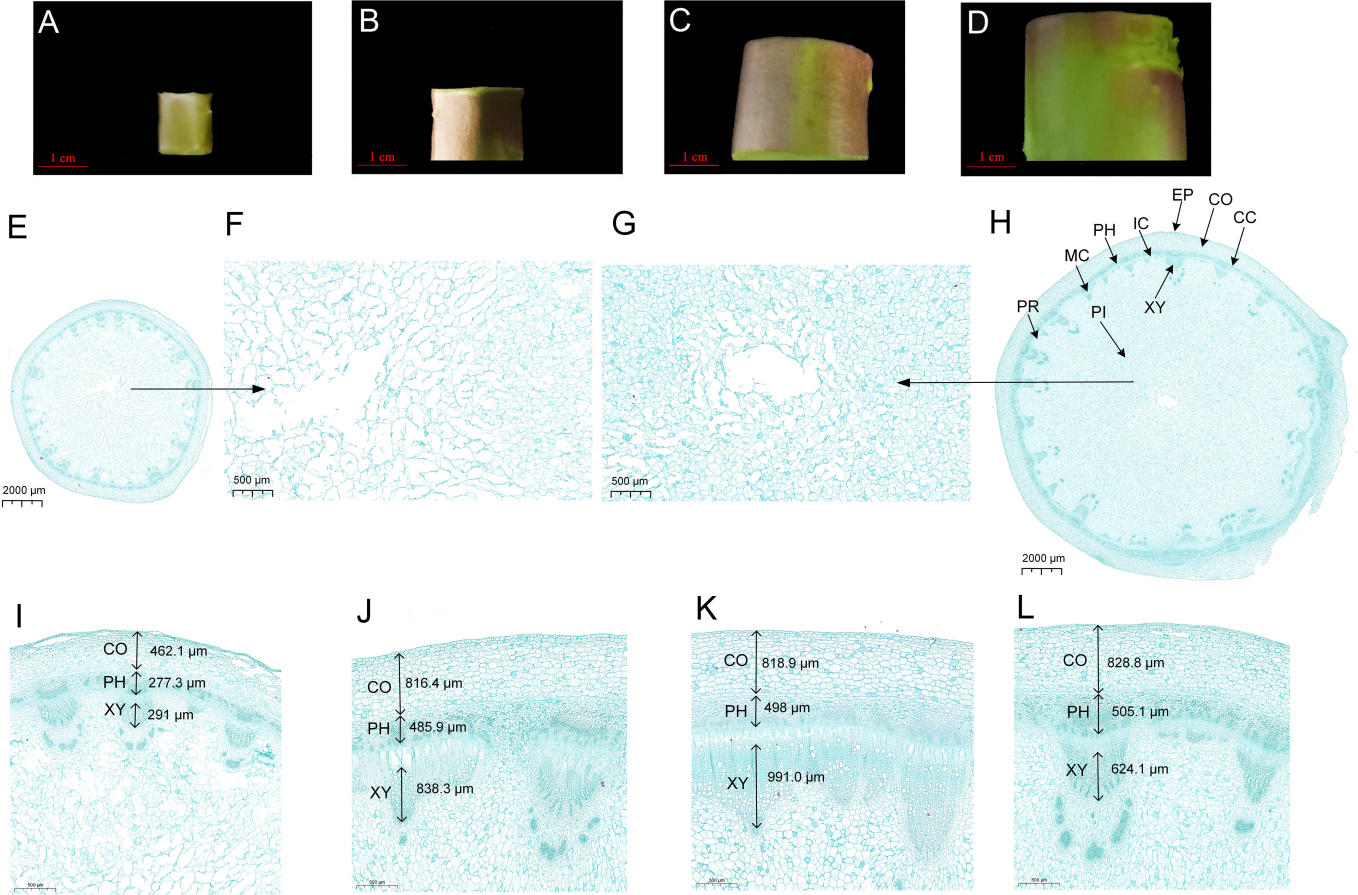
Anatomical analysis of lettuce fleshy stem at different expansion stages (S1, S2, S3, and S4). **(A–D)** The transverse diameters are 1 cm (S1 stage), 2 cm (S2 stage), 3 cm (S3 stage), and 4 cm (S4 stage), respectively, **(E)** Global cross-sections of S1 stage, **(F)** Local maps of medullary cells at S1 stage, **(G)** Local maps of medullary cells at S4 stage, **(H)** Global cross-sections at S4 stage, **(I–L)** Local maps of the epidermis to vascular bundles from S1 to S4 stages. EP, Epidermis; CO, Cortex; CC, Cork Cambium; PH, Phloem; PR, Pith Ray; IC, Interfascicular Cambium; MC, Midbundle Cambium; XY, Xylem; PI, Pith.

The anatomical structure analysis of lettuce fleshy stem displayed that the development of the fleshy stem was mainly caused by the occurrence and activity of vascular cambium (**Figure 1**). The lettuce fleshy stem comprised a cortex, vascular bundle, and pith from outside to inside; the vascular bundle contained phloem, cambium, and xylem (**Figures 1E–H**). Vascular bundles arranged in a ring on the inside of the cortex, and the pith rays were located between the vascular bundles, which connected the cortex and the medulla (**Figure 1H**). As displayed in **Figures 1I, J**, the cell volume increased significantly from S1 stage to S2 stage; and the cambium parenchyma cells divided tangentially, inducing a rapid increase in the diameter of the fleshy stem. Compared to S1 stage, the cortex, phloem, and xylem at S4 stage increased about two, two, and three times (**Figure 1L**). The transverse diameter of the xylem was larger than that of the phloem and increased more rapidly (**Figures 1I–L**). The pith cells at S1 stage were loosely arranged and numerous gaps existed between the pith cells. During the process of stem expansion, the number of pith cells increased rapidly and the pith cells were arranged very closely, which might be one of the main reasons to cause a thickening of the fleshy stem (**Figures 1F, G**).

### Metabolomics analysis of lettuce fleshy stem

**Figure 2:**
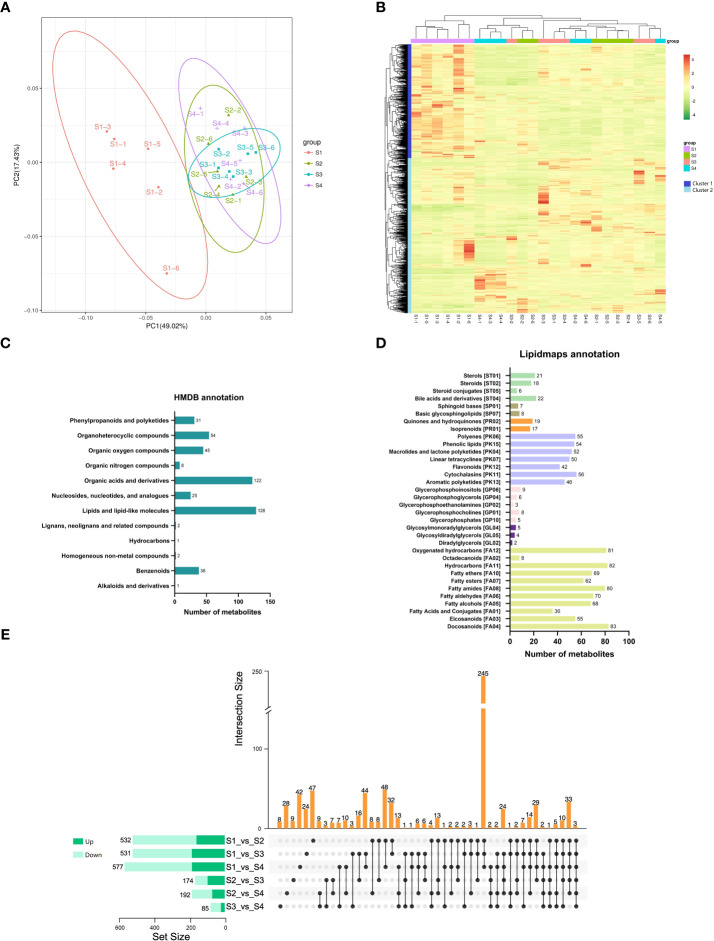
Metabolic analysis of lettuce fleshy stem during four expansion stages (S1, S2, S3, and S4). **(A)** Principal component analysis (PCA) of the identified metabolites in four expansion stages (S1, S2, S3, and S4). **(B)** Heatmaps of all metabolites during the four expansion stages (S1, S2, S3, and S4). **(C)** Taxonomic annotations of metabolites by HMDB database. **(D)** Taxonomic annotations of metabolites by LIPIDMAPS database. **(E)** Comparisons of up-regulated metabolites (dark green) and down-regulated metabolites (light green) in the paired comparison of each expansion period.

To investigate the metabolite changes during the fleshy stem expansion process, metabolomics analysis of S1, S2, S3, and S4 stages was conducted. PCA analysis showed that the first two principal components (PC1 and PC2) separated 24 samples, accounting for 49.02% and 17.43% of the total variability, respectively (**Figure 2A**). In addition, the samples on the PCA analysis map were clustered into four groups. The metabolite of S1 stage was mainly at the negative end of PC1; while, the metabolite of S2, S3, and S4 stages were mainly at the positive end of PC1. The metabolites of S2, S3, and S4 stages were similar, even with a large part of overlap. The results indicated that different metabolites existed in the expansion process of lettuce fleshy stem. A total of 1,251 metabolites were detected and clustered into two categories (cluster 1 and cluster 2) during the four expansion stages (S1, S2, S3, and S4 stages). The metabolite in cluster 1 highly expressed in S1 stage and the metabolite in cluster 2 mainly expressed in S2, S3, and S4 stages (**Figure 2B**).

The identified metabolites were annotated using the HMDB and LIPIDMAPS databases. As shown in **Figure 2C**, 457 metabolites were classified into twelve categories by the HMDB database. The metabolite belonged to lipids and lipid-like molecules were the most abundant (128 metabolites), followed by organic acids and derivatives (122 metabolites). In the LIPIDMAPS annotation analysis, the most metabolites belonged to Docosanoids (83 metabolites), followed by Hydrocarbons (82 metabolites) and Fatty amides (80 metabolites) (**Figure 2D**). The metabolites identified in S1, S2, S3, and S4 stages were compared between groups, and they were identified by VIP, FC, and *p*-value (FC > 1.0, *p* value < 0.05, and VIP > 1.0). The pairwise comparisons of the identified metabolites among the four stages are depicted in **Figure 2E**. The three comparison pairs of S1 vs. S2, S1 vs. S3, and S1 vs. S4 contained the most differentially expressed metabolites (DEMs) (532, 531, and 577 DEMs, respectively). The comparison pairs of S3 vs. S4 had the least metabolites (85 DEMs), indicating the similarity between these two stages. Only three DEMs were identified at all the four expansion stages (S1, S2, S3, and S4). A total of 245 DEMs were identified to exist in the three combinations of S1 vs. S2, S1 vs. S3, and S1 vs. S4, indicating that the development of S2, S3, and S4 stages was similar.

### Transcriptome analysis of lettuce fleshy stem

**Figure 3:**
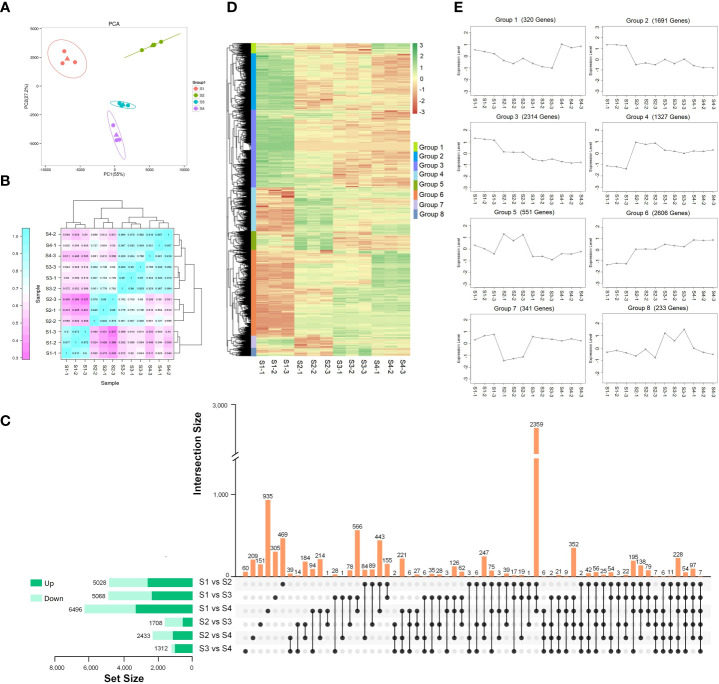
Transcriptome analysis of lettuce fleshy stem during four expansion stages (S1, S2, S3, and S4). **(A)** Principal component analysis (PCA) of gene expression at four expansion stages (S1, S2, S3, and S4). **(B)** Pearson correlation coefficient of gene expression in four expansion stages (S1, S2, S3, and S4). **(C)** Comparisons of up-regulated DEGs (dark green) and down-regulated DEGs (light green) in the paired comparison of each expansion period. **(D)** Hierarchical clustering heatmap of the identified DEGs. **(E)** K-means clustering of the DEGs expression.

To investigate the DEGs during different expansion stage, transcriptome analysis of S1, S2, S3, and S4 stages with three biological replicates was conducted. The cDNA libraries were constructed and sequenced using the Illumina HiSeq 4000 platform. A total of 596,863,836 raw reads were generated from 12 samples, and 298,431,918 high-quality clean reads were identified after filtration. The average percentage of Q20 and Q30 bases was 97.74% and 93.46%, respectively. The percentage of GC was more than 43.65%. The clean reads were mapped to the lettuce genome, with an average efficiency of 94.33% (**Table S2**). A total of 42,916 transcripts were detected and the expression level of each gene was normalized by FPKM. The identified transcripts among four expansion stages (S1, S2, S3, and S4) were analyzed by PCA analysis. As shown in **Figure 3A**, the gene expression of S1 and S2 stages were located at the negative and positive ends of PC1, respectively. The gene expression of S3 and S4 stage were both located at the negative end of PC2, indicating the similarity between S3 and S4 stage. The correlation of gene expression levels was higher in the three biological replicates of the same stage. The gene expression level at S3 stage showed high correlation those at S2 and S4 stages. While, The gene expression level at S1 stage showed the lowest correlations with S2, S3, and S4 stages (**Figure 3B**).

The DEGs in the six comparison pairs (S1 vs. S2, S1 vs. S3, S1 vs. S4, S2 vs. S3, S2 vs. S4, S3 vs. S4) were analyzed (**Figure 3C**). Compared with the S1 stage, there were 5,028 (2,727 up-regulated DEGs and 2,301 down-regulated DEGs), 5,068 (2,478 up-regulated DEGs and 2,590 down-regulated DEGs), and 6,496 DEGs (3,446 up-regulated DEGs and 3,050 down-regulated DEGs) existed in S2, S3, and S4 stages, respectively. The results indicated that the development of fleshy stem at S2, S3, and S4 stages was significantly different with S1 stage. Only seven genes were co-expressed among the six comparison pairs (S1 vs. S2, S1 vs. S3, S1 vs. S4, S2 vs. S3, S2 vs. S4, S3 vs. S4). There were 2,359 co-expressed genes in the comparison pairs of S1 vs. S2, S1 vs.S3, and S1 vs. S4. Among the individual comparison pairs, a total of 935 DEGs were identified in the comparison pair of S1 vs. S4, indicating the largest difference existed in S1 and S4 stage. In the comparison of S3 vs. S4, 60 genes were identified, indicating the smallest difference existed in S3 and S4 stage. All the results showed that the development of fleshy stem in S2, S3, and S4 stages was significantly different from S1 stage.

A total of 9,383 DEGs were identified after merging the differential genes of all combinations, and a hierarchical clustering heatmap was drawn using the standardized FPKM Z-score value (**Figures 3D, E**). These DEGs were divided into eight groups (1-8). The 320 genes in group 1 showed highly expressed at S4 stage; 1,691 genes in group 2 were highly expressed at S1 stage. The 2,314 genes existed in group 3 showed gradually decreased expression during fleshy stem expansion process. The expression of genes in group 4 increased rapidly at S2 stage, then decreased slightly, but the overall expression was higher than those at S1 stage. 551 genes in group 5 showed higher expression at S2 stage. Group 6 contained the most number of genes (2,606), and the expression levels of these genes increased gradually with the expansion of the stem. The expression of 341 genes in group 7 was the lowest at S2 stage; and the expression of 233 genes in group 8 was the highest at S3 stage. In addition, most genes showed significant changes at S2 stage in these eight groups, indicating that S2 stage was very important for fleshy stem expansion.

### Transcription factor (TF) family analysis

**Figure 4:**
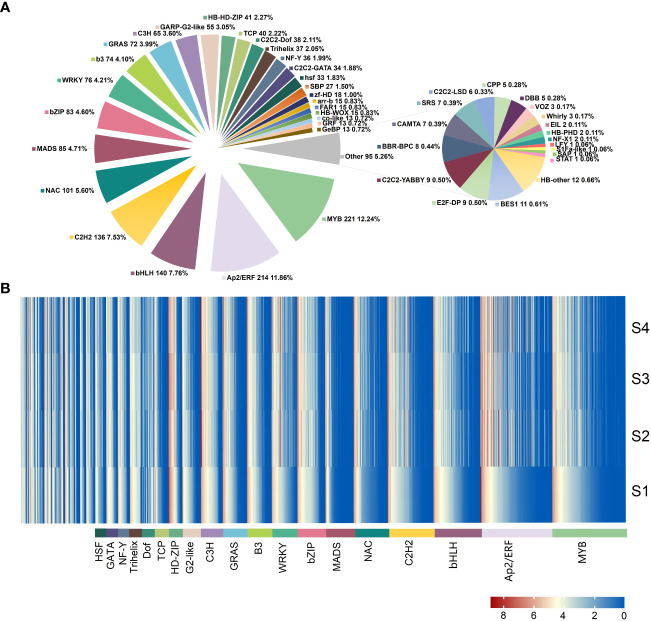
Transcription factor family analysis. **(A)** Summary of transcription factor families, the pie chart showed the number and proportion of each transcription factor family; **(B)** Expression pattern analysis of transcription factor family genes using heatmap. The data were calculated using log2 (FPKM+1).

A total of 1,805 DEGs were identified as TFs by the further analysis, and these genes belonged to 46 TF families, including MYB, bHLH, AP2, and C2H2 (**Figure 4A**, **Table S3**). The expression levels of identified TF family gene were showed by heatmap (**Figure 4B**). The top 3 TF families with the highest number of genes were MYB (221), AP2 (214), and bHLH (140), with high overall expression in the four stages. The expression of some genes belonged to WRKY, MYB, and AP2/ERF TFs was higher at S1 stage and then decreased at S2 and S3 stages, indicating that these genes might play a regulatory role in the early stage of lettuce fleshy stem expansion. The expression of some TF genes such as bZIP and HSF was low at S1 stage but increased at S2 and S3 stages. PHD and NF-YC TF genes were not significantly expressed at S1 and S2 stages but up-regulated at S3 stage. These results suggested that the TFs played important regulatory roles during different expansion stages of lettuce fleshy stem.

### Enrichment analyses of the transcriptome and metabolome

**Figure 5:**
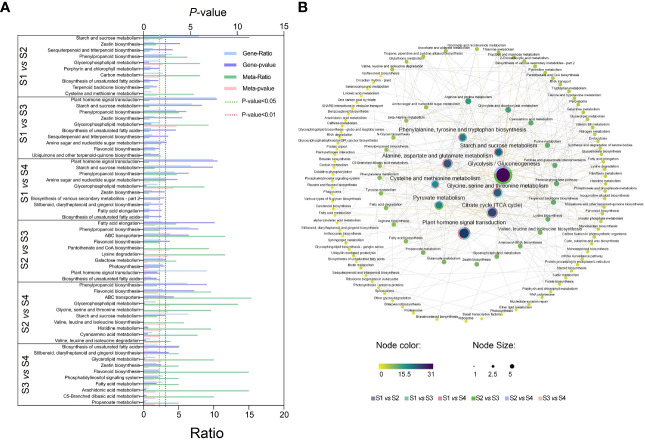
KEGG pathway annotated analysis in different lettuce stem expansion stages (S1, S2, S3, and S4 stages). **(A)** The top 10 pathways associated with transcriptome and metabolome. Ratio: the number of differential metabolites or differential genes enriched in this pathway/the number of metabolites or genes annotated in this pathway. **(B)** Correlation analysis of each pathway. Different dots represented different metabolic pathways; yellow to purple indicated the degree of enrichment in the whole expansion stage, and the central fan chart displayed the degree of enrichment of the pathway in each comparison group. The data were calculated by -log10 (*p*-value), and the higher the value, the greater the enrichment.

Significant enriched metabolic pathways related to DEGs and DEMs were identified by KEGG enrichment analysis (**Figure 5A** and **Table S4**). A total of 33 metabolic pathways were identified from the top 10 significantly enriched metabolic pathways for all six comparisons. The highly expressed metabolic pathways in the transcriptome and metabolome were ‘Starch and sucrose metabolism’, ‘phenylpropanoid biosynthesis’, and ‘Plant hormone signal transduction’. The most significantly expressed pathways in transcriptome were ‘Phenylpropanoid biosynthesis’, ‘Starch and sucrose metabolism’, ‘Zeatin biosynthesis’, and ‘Flavonoid biosynthesis’. The most significantly expressed pathways in the metabolome were ‘Glycerophospholipid metabolism’, ‘Carbon metabolism’, ‘Pantothenate and CoA biosynthesis’, and ‘Glycine, serine, and threonine metabolism’. The pathways related to the growth and development of lettuce fleshy stem were mainly ‘Starch and sucrose metabolism’, ‘Zeatin biosynthesis’, and ‘Plant hormone signal transduction’ by the comprehensive analysis, which were related to the physiological processes of sugar and acid metabolism as well as hormone synthesis.

In order to further analyze the correlation between the various pathways, the association maps of the significantly enriched pathways in the six combinations were drawn (**Figure 5B**). A total of 119 different pathways were enriched. The pathways, which closely related to lettuce growth, were located in the middle of the figure. The glycolytic synthesis pathway had a central role in all pathways. The glycolytic synthesis pathway directly related to multiple significant enrichment pathways. First, glycolysis pathway directly connected starch and sucrose metabolism, citrate cycle (also known as TCA cycle), as well as various amino acid synthesis pathways. Glycolysis pathway also participated in the synthesis and metabolism of chlorophyll, endogenous hormones, alkaloids, and other substances, which provided a material basis for lettuce growth and development. Second, glycolysis pathway could regulate the synthesis of plant hormones in lettuce through hormone signal transduction pathways. The differential pathways of S1 vs. S2, S1 vs. S3, and S1 vs. S4 accounted for more than half of the total, indicating that the metabolic transcription differences in S2, S3, and S4 stages are important factors in lettuce stem expansion.

### Analysis of the sugar acid synthesis metabolic pathway

**Figure 6:**
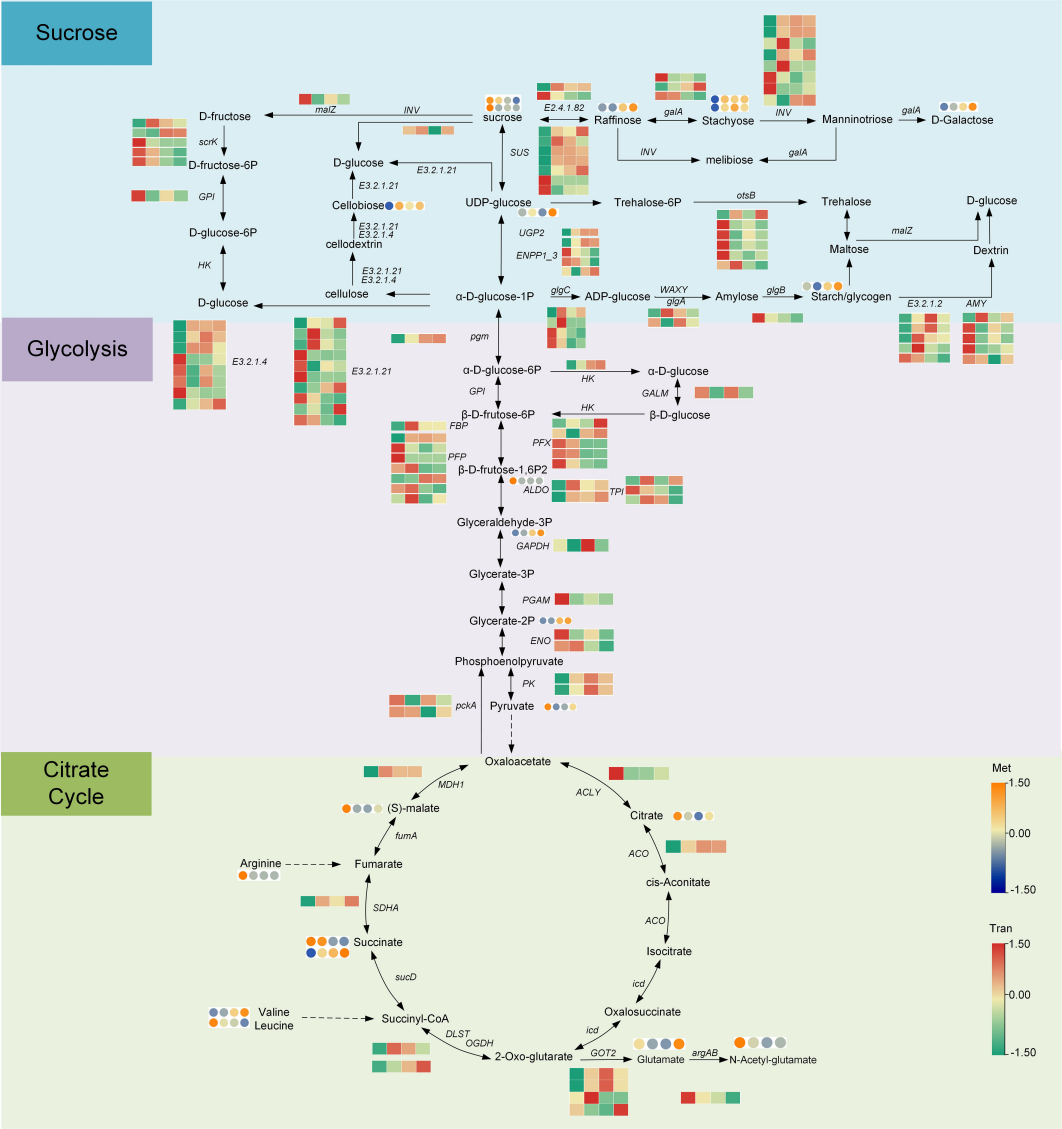
Analysis of genes and metabolites expressed in the sugar and acid biosynthesis pathways during lettuce fleshy stem expansion stages (S1, S2, S3, and S4). The red (up-regulated) and green (down-regulated) in the heatmap represent gene expression, and the yellow (up-regulated) and blue (down-regulated) in the heatmap represent the expression of metabolites. The expression level was calculated using log2(FPKM+1).

The starch and sucrose metabolism pathways were identified through the transcriptomic and metabolomics pathway analysis. As shown in **Figure 6** and **Tables S5**-**S6**, in the process of starch and sucrose metabolism, most enzyme genes (including 19 enzymes, 73 enzyme genes, and 24 metabolites) showed an upward trend during fleshy stem expansion stages. Compared with S1 stage, the expression of some genes, encoding sucrose synthase (SUS), UTP–glucose-1-phosphate fructofuranosidase (UGP2), fructofuranosidase (INV), endoglucanase (E3.2.1.4), and raffinose synthase (E2.4.1.82), increased five-fold at S2, S3, and S4 stages. However, the expression patterns of some enzyme genes encoding fructokinase (scrK), gluc-1-phosphate adenylyltransferase (glgC), 1,4-alpha-glucan branching enzyme (glgB), and beta-amylase (E3.2.1.2) decreased in lettuce stem expansion process. The content of some metabolites, such as raffinose, stachyose, starch, and cellobiose, was low at S1 stage, but increased at S3 and S4 stages. The content of sucrose decreased in the fleshy stem expansion process. The content of sucrose decreased two-fold at S4 stage compared with S1 stage, indicating that sucrose began to accumulate at the beginning of the expansion for the later lettuce expansion.

The expression of most enzyme genes in the glycolysis pathway (including 9 enzymes, 30 enzyme genes, and 10 metabolites) increased gradually during expansion stages. Compared to S1 stage, the expression of genes, encoding some enzymes such as phosphoglucomutase (pgm), hexokinase (HK), class I (ALDO), and triosephosphate isomerase (TPI) increased ten-fold at S2, S3, and S4 stages. However, the expression of some genes, encoding 6-phosphofructokinase 1 (PFK), phosphoenolpyruvate carboxykinase ATP (pckA), and enolase (ENO), decreased at S2, S3, and S4 stages. The expression of most enzyme genes in the TCA cycle such as aconitate hydratase (ACO), flavoprotein subunit (SDHA), and malate dehydrogenase (MDH1) was the lowest at S1 stage but increased two-fold at S2, S3, and S4 stages. Overall, most metabolites and enzyme genes involved in sugar synthesis, glycolysis, and the citrate cycle were up-regulated during the process of fleshy stem expansion.

### Analysis of the hormone synthesis metabolic pathway

**Figure 7:**
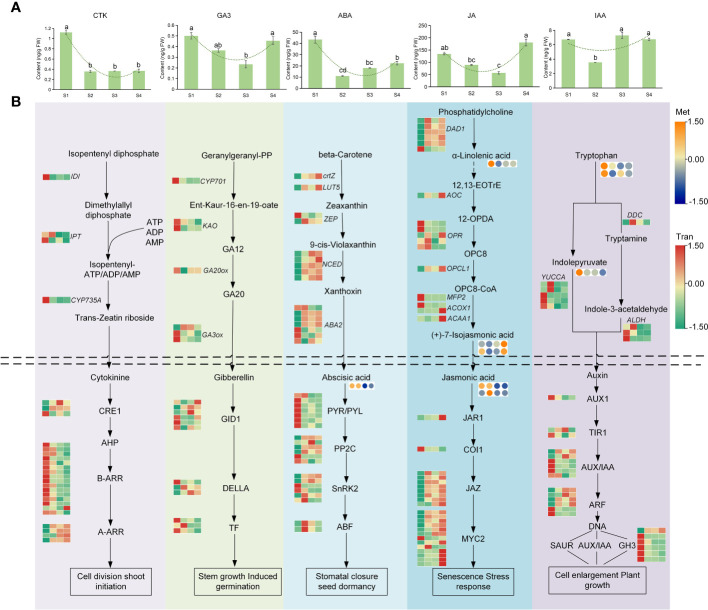
The anabolism of plant hormone during lettuce fleshy stem expansion stages (S1, S2, S3, and S4). **(A)** The contents of CTK, GA, ABA, JA, and IAA in the four expansion stages (S1, S2, S3, and S4). Lowercase letters **(A–D)** in each column denote significant differences. **(B)** Analysis of the synthetic metabolic pathway of CTK, GA, ABA, JA, and IAA in the four stages (S1, S2, S3, and S4). Above the dotted line is the synthetic pathway, and below the dotted line is the metabolic pathway. The red (up-regulated) and green (down-regulated) in the heatmap represented gene expression, and the yellow (up-regulated) and blue (down-regulated) in the heatmap represented the metabolite accumulation.

Plant hormones play important roles in the process of plant organ expansion. The hormone pathways, including CTK, IAA, GA, JA, and ABA, were analyzed to explore its roles in lettuce fleshy stem expansion stages (**Figure 7**, **Tables S5**-**S6**). In CTK, the overall expression level of genes encoding enzymes was high at S1 stage and decreased at S2, S3, and S4 stages. The expression of some genes encoding isopentenyl-diphosphate Delta-isomerase (IDI), adenylate dimethylallyltransferase (IPT), and cytokinin trans-hydroxylase (CYP735A) decreased two-fold at S2, S3, and S4 stages. Cytokinin content also showed a two-fold decrease at S2, S3, and S4 stages than at S1 stage. The expression profiles of genes such as *CYP701* and phytochrome-interacting factor 4 involved in GA pathway, were higher at S1 stage and lower at S2, S3, and S4 stages. However, the expression of some genes encoding GA20ox, GA receptor GID1, and DELLA protein, was high at S1 stage and decreased at S2 stage but showed an upward trend at S3 and S4 stages. The GA content initially decreased and then increased, which showed similar results to the gene expression trend.

In ABA pathway, the expression of genes before ABA synthesis was low at S1 stage, and then gradually increased with lettuce expansion process. For example, compared to S1 stage, the expression levels of *LUT5* and *NCED* increased by 2-15 times at S4 stage. The expression of genes such as the ABA receptor PYR/PYL family gene in ABA metabolism pathway highly expressed at S1 stage and decreased at S2, S3, and S4 stages. The ABA content was highest at S1 stage and reduced two-fold at S2, S3, and S4 stages, which was similar to the trend of gene expression. In the process of JA synthesis, most genes were highly expressed at S1 or S4 stage, such as *OPR* and *ACOX1*. In the process of JA metabolism, the expression of genes, such as *JAR1*, *JAZ*, and *MYC2*, was higher at S2, S3, and S4 stages. The JA content identified by metabolome was higher at S1 and S2 stages than at S3 and S4 stages, which might related to the high expression of JA metabolism genes at S4 stage. The overall trend of JA content at different expansion stages was similar to in result of metabolome.

The expression patterns of most genes in the auxin synthesis pathway, were highly expressed at S1 stage, such as aldehyde dehydrogenase *NAD+*(*ALDH*) and *YUCCA*. The expression profiles of most genes in the metabolic pathway of auxin were similar to the expression patterns of synthetic genes; and the highest expression levels of genes such as *AUX1*, *TIR1*, and auxin-responsive protein IAA, were identified at S1 stage, which indicated that auxin had a fast metabolic transformation process in the early growth stage. In summary, during lettuce stem expansion process, the overall expression of CTK gradually decreased, whereas GA, ABA, IAA, and JA decreased first and then increased.

### Correlation network analysis of anabolism pathways and gene verification

**Figure 8:**
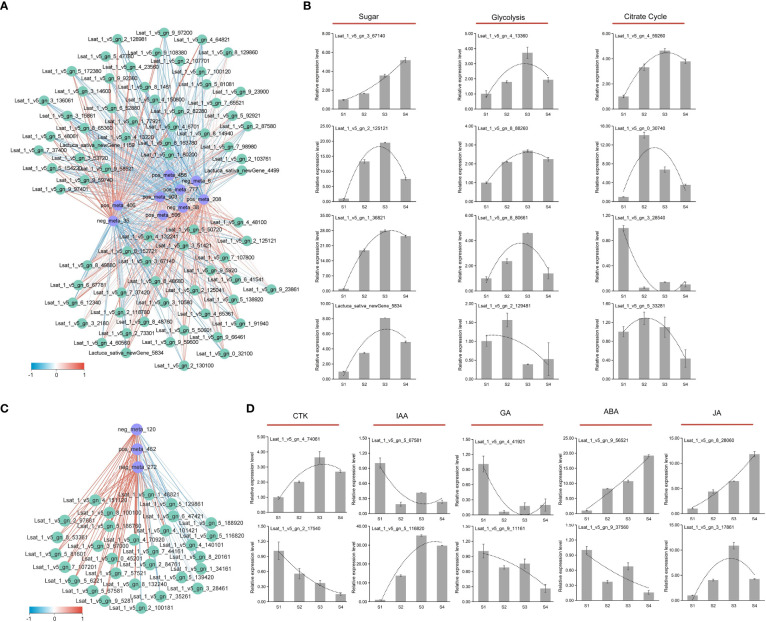
Correlation network analysis and gene verification of sugar, acid, and hormone metabolism. **(A)** Correlation network analysis diagram of DEMs (blue) and DEGs (green) related to glucose synthesis metabolism. The correlation was represented by blue and red lines from low to high. **(B)** qRT-PCR analysis of genes involved in sugar and acid biosynthesis. **(C)** The correlation network analysis diagram of DEMs (blue) and DEGs (green) related to auxin synthesis metabolism. The correlation was represented by blue and red lines from low to high. **(D)** qRT-PCR analysis of genes involved in hormone synthesis.

The sugar-acid synthesis correlation network analysis showed the correlation between sucrose and other metabolites was the complete opposite (**Figure 8A**). Sucrose showed a significant positive correlation with most genes in the pathway, while other metabolites depicted a significant negative correlation with these genes. To verify the validity of the transcripts, twelve genes in the sugar synthesis pathway were selected for qRT-PCR analysis (**Figure 8B**). The expression of *Lsat_1_v5_gn_3_67140*, *Lsat_1_v5_gn_2_125121*, *Lat_1_v5_gn_1_36821*, and *Lactuca_ sativa_newGene_5834* involved in the sugar synthesis process, showed an overall upward trend in both the four stages (S1, S2, S3, and S4). In the glycolysis pathway, *Lsat_1_v5_gn_4_13360*, *Lsat_1_v5_gn_8_88260*, and *Lsat_1_v5_gn_8_80661* showed an overall upward trend; and the overall expression of these genes at S2, S3, S4 stages increased about two-four times than at S1 stage. In the late stage of glycolysis, the expression level of *Lsat_1_v5_gn_2_129481* showed a downward trend, and the expression level at S4 stage decreased about twice compared with S1 stage. In Citrate Cycle, the expression levels of *Lsat_1_v5_gn_4_59260* and *Lsat_1_v5_gn_0_30740* both increased at S2, S3, and S4 stages compared with S1 stage, but *Lsat_ 1_v5_gn_3_28540* and *Lsat_1_v5_gn_5_33281* showed a downward trend. The qRT-PCR results of genes in the sugar and acid synthesis metabolic pathways were generally consistent with the trend in transcriptome data, indicating that the reliability of transcript data was high.

The strong positive correlation between metabolites and genes involved in IAA biosynthetic pathway was identified by the correlation network analysis (**Figure 8C**). Tryptophan and pyruvate had a high positive correlation with most genes, reaching 0.9. To verify the validity of the transcript, genes involved in hormone pathway (CTK, IAA, GA, JA, and ABA) were selected for qRT-PCR analysis (**Figure 8D**). In the CTK synthesis pathway, the expression of *Lsat_1_v5_gn_2_17540* depicted a downward trend during the fleshy stem expansion process. In the CTK metabolic pathway, the expression of *Lsat_1_v5_gn_4_74061* showed an upward trend during the fleshy stem expansion process. In the GA synthesis metabolic pathway, the expression levels of *Lsat_1_v5_gn_4_41921* and *Lsat_1_v5_gn_9_11161* decreased at S2, S3, and S4 stages than at S1 stage. In the ABA synthesis pathway, *Lsat_1_v5_gn_9_56521* depicted an upward trend expression at S2, S3, and S4 stages compared to S1 stage. In the ABA metabolic pathway, the expression of *Lsat_1_v5_gn_9_37560* showed a downward trend. In the JA synthesis pathway, the expression levels of *Lsat_1_v5_gn_8_28060* and *Lsat_1_v5_gn_3_17861* increased at S2, S3, and S4 stages than at S1 stage. Compared with the S1 stage, the expression level of *Lsat_1_v5_gn_5_116820*, which belonged to auxin metabolic pathway, increased about ten times or more at S2, S3, and S4 stages. The expression patterns of genes involved in hormone synthesis metabolic pathway identified by qRT-PCR were generally consistent with the trend in transcriptome data, indicating that the reliability of transcript data was high.

## Discussion

‘Omics’ technologies, such as metabolomics, transcriptomics, and proteomics have been widely applied to identify a series of molecular mechanism involved in the process of plant’ growth and development or the interaction between plants and external factor (Wang et al., 2021a; Li et al., 2022; Lin et al., 2022). As one of the most popular vegetables, domesticated lettuce contained some horticultural types such as butterhead, loose leaf, and stem lettuce. The molecular mechanism involved in leaf development in lettuce has been explored. Lettuce *LsKN1* has been identified to generate wavy leaves by regulating plant hormone signaling pathways (Jia et al., 2022; Wang et al., 2022). The genes regulating anthocyanin in lettuce has also been analyzed (Su et al., 2022). The domestication history and domestication-shaped genetic architecture of cultivated lettuce have been elucidated, which provides valuable genomic resources for cultivated lettuce breeding (Zhang et al., 2020; Wei et al., 2021). However, the metabolome and transcriptome combined analysis exposed to fleshy stem expansion is not common.

**Figure 9:**
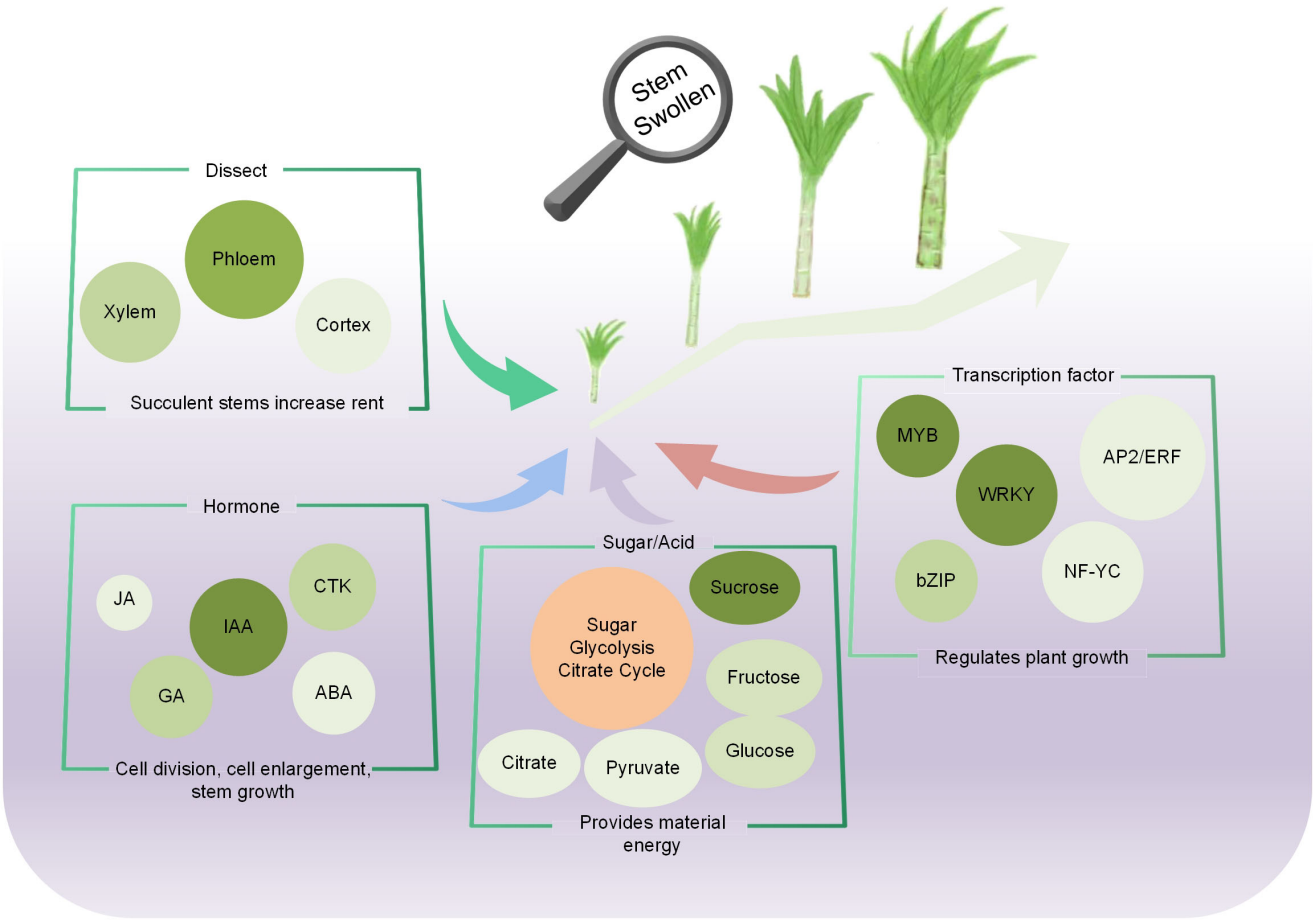
Regulation pattern of the fleshy stem expansion in stem lettuce.

The growth and expansion of lettuce fleshy stem is a complex dynamic process coordinated by many regulatory elements, including the activity of cells, plant hormones, metabolites, and gene regulation. In the study, the changes in morphology, physiology, metabolites, and gene expression during lettuce fleshy stem expansion stages (S1, S2, S3, and S4) were identified. The occurrence and activity of the vascular cambium led to the development of the fleshy stem. Metabolome and transcriptome analyses revealed that the pathways of sugar synthesis, glycolysis, TCA cycle, and plant hormone anabolism played important roles during fleshy stem expansion. Plant hormone such as IAA, GA, and JA showed different promoting effects, while ABA exerted inhibitory effects. Sugar biosynthesis and metabolism gradually increased during lettuce stem expansion. TFs also played an important role in the fleshy stem expansion process. For example, MYB, bHLH, and bZIP changed significantly with lettuce expansion (**Figure 9**).

The distribution and allocation of photosynthate in source-sink units is of great significance to the formation of the vegetative organs (Tang et al., 2021). In cucumber, *CsAGA2* decomposed RFOs into hexose, and transported it to pulp cells through the apoplast, promoting the accumulation of sugar in fruit (Liu et al., 2022). The increase in the size and number of parenchyma cells in the cortex and medulla was the main reason for stem development (Kolomiets et al., 2001; Zhong et al., 2007). Hormone signal transduction, sugar starch metabolism, and phenylpropanoid synthesis metabolism were enriched in the main root enlargement of *Panax notoginseng* (Li et al., 2019). During the process of fleshy stem expansion, the photosynthate was rapidly distributed to the fleshy stem; cells divided inward to rapidly increase the internal diameter of the fleshy stem. At the same time, the pith cells were arranged tightly. Sugar synthesis, glycolysis, TCA cycle, and plant hormone anabolism were identified to play important roles in the fleshy stem expansion process in stem lettuce.

As an energy substance for plant growth and development, sugar involve in plant growth and development as a signaling substance (Peng et al., 2018). Sucrose played a key role in integrating developmental stages and environmental cues to regulate plant yield (Fernie et al., 2020; Liao et al., 2020; Wu et al., 2021; Ren et al., 2022). In this study, the sucrose content was highest in the early stage of stem expansion in lettuce, indicating that it stored a large amount of energy in the early stage for the later stem expansion. In the later stage of lettuce expansion, sucrose was converted into glucose and fructose, which might also explain the increased glucose and fructose content and the decrease of sucrose in the later stage. Hormones have been identified to be essential endogenous substances for plant growth and development (Liang et al., 2014; Miyashima et al., 2019). GA was considered as a potentially important regulator of cell elongation and expansion in plants (Ayano et al., 2014). Some GA-related genes were differentially expressed in different stages of carrot root development (Xu et al., 2014; Wang et al., 2015). Auxin could stimulate GA biosynthesis, and exogenous use of 2,4-D could stimulate the expression of *GA20ox* and *GA3ox* (Cong et al., 2019). CTKs increased cell division in Arabidopsis, tomato, and tobacco and were also associated with cell proliferation in the early stages of tuber growth (Katsarou et al., 2016). ABA and JA could involve in many processes regulating plant growth and development. The β-glucosidase gene (*ClBG1*) was a key gene in regulating ABA content, reducing the seed size of watermelons (Wang et al., 2021b). At different stages of tulip bulb development, the lipoxygenase genes *TgLOX4* and *TgLOX5* involved in the biosynthesis of JA; and the silencing of *TgLOX4*, and *TgLOX5* genes inhibited the growth of tulip bulbs (Sun et al., 2022). In the study, the content of GA in lettuce fleshy stems was the highest in the early stage of stem expansion, and then decreased with expansion; and the related genes involved in GA pathway also demonstrated a similar trend. These results indicated that GA might play an important role in cell proliferation in the early growth and development of lettuce. The content of CTK and ABA was the highest in the early stage of lettuce expansion, gradually decreasing with expansion, indicating that CTK and ABA played an important role in the early stage of lettuce expansion. However, the content of JA was higher than that of other hormones, indicating that JA played a promoting role during lettuce stem expansion.

Plant growth and development are not only regulated by enzyme genes but also by transcription factors (Smet et al., 2019). The bHLH TF gene *TMO5* was expressed in the early provascular initial cells. The heterodimer TMO5/LHW formed by TMO5 and LHW could directly target and induce the expression of *LOG4* and *LOG3* to control cell proliferation (De Rybel et al., 2013; De Rybel et al., 2014). *IbNAC083* was the core initiation factor for storage root development (He et al., 2021). Several WRKYIII TF genes showed different expression patterns at stem expansion stages in lettuce (Du et al., 2022). Overall, transcription factors might participate in the key biological processes during fleshy stem expansion. Studying the molecular mechanism of specific TFs is of great significance for regulating the growth and expansion of lettuce stems. The function of transcription factors involved in controlling stem expansion should be analyzed in-depth to improve the cultivation of high-yield lettuce. In the study, a comprehensive analysis of global changes in the metabolites and gene expression was conducted during fleshy stem expansion in stem lettuce. The important genes and metabolites identified in the progress of lettuce fleshy stem expansion will provide important information for the further analysis of the molecular regulatory mechanisms during the process of lettuce stem growth and development.

## Data Availability

The datasets presented in this study can be found in NCBI, the accession number is PRJNA844256.
