# Supplementary material for: Combined analysis of the transcriptome and metabolome provides insights into the fleshy stem expansion mechanism in stem lettuce
Source: Front Plant Sci. 2022 Dec 15;13:1101199. doi: 10.3389/fpls.2022.1101199 (PMC9798005; doi:10.3389/fpls.2022.1101199)
Supplement: Supplementary file 1 [file Table_1.docx]

**Table S1** Sequences of primers used in qRT-PCR.

| **Gene ID** | **Forward primer (5'→3')** | **Reverse primer (5'→3')** |
| --- | --- | --- |
| *Lsat_1_v5_gn_3_67140* | GGCGAAGCACAGCATGTTGAATG | CCATCCATCCTTGCCAGATACTCCT |
| *Lsat_1_v5_gn_2_125121* | CGTCAATTCGTCAGATCCACTCCTT | GAAGTACCTCCTCGTTCAGCACAA |
| *Lsat_1_v5_gn_1_36821* | GGACACGGTCGTAGCAATGGA | AGTGAGAGGCGGCGAGAAGA |
| *Lactuca_sativa_newGene_5834* | TTGGACGGTTGGATTGATTCTAGCA | TGACTGATGGTATCGGCGGAAGA |
| *Lsat_1_v5_gn_4_13360* | CCTTCCGATTCTGTTGCCATCATTG | AGTCCAGCATCCATCAAGTTACCAA |
| *Lsat_1_v5_gn_8_88260* | CTTCTTGCTCAGAGTGCGGTTCA | GGCGGTGCTTCATCCATAATAGACT |
| *Lsat_1_v5_gn_8_80661* | TCCGTCATCTTCGTGCTCTCCAT | TTCCACTTCGTCTACGCTTCCAATG |
| *Lsat_1_v5_gn_2_129481* | AGCCGTGGTAATCCTACAGTTGAAG | AAGGCAGGTCCGATTATTGAGTTG |
| *Lsat_1_v5_gn_4_59260* | GTGGTGACTTGGTTGATGCTGTTG | TTGGAGGTGGTGGAGAGGATACTT |
| *Lsat_1_v5_gn_0_30740* | CGCAGAGGTATTTCCAGGCTTCAAA | CGCAGATTCCTTCACGGCAAGAT |
| *Lsat_1_v5_gn_3_28540* | GCTCCACTTGTTGCCACTCTCC | GATGACACTAGCACCACCACCAG |
| *Lsat_1_v5_gn_5_33281* | AGGTAGCGGTATCTCTTCTTCTCCA | CCTTCCTCTGTACGATAAGCACCAA |
| *Lsat_1_v5_gn_4_74061* | GCGGAAGCAGAGCCTTACAGTAT | CGATGCGTGTTAGAATGTTCTCAGA |
| *Lsat_1_v5_gn_2_17540* | CCTGCTGAAGATGTTCCAGTTGATG | CGTATCTAATGTCCGCCACCTCAT |
| *Lsat_1_v5_gn_5_67581* | GCACGGAATCCAAAGGCAGTTC | TCACCAACAAGCATCCAATCGC |
| *Lsat_1_v5_gn_5_116820* | ATTCTCCGACTGACGCCACCA | CATTCTCCACCGCCGATTGTAACT |
| *Lsat_1_v5_gn_4_41921* | AAGTTGACCGCAGCACCAGTAA | CGCCGTAGTTAAGCACCGTGTA |
| *Lsat_1_v5_gn_9_11161* | GAAGAGGAACCAAGCCAACCTAAGT | GTAGCATTCTGTGTCGTCAGTGTCT |
| *Lsat_1_v5_gn_9_56521* | AATCGCCGTCGCTTCCAGTTC | GCCGCTAGGTCCAGGATTGTTAC |
| *Lsat_1_v5_gn_9_37560* | GATCCAACAGATCAACGCTCCAATC | GAAGAGTGGTGACGGAACGGTAG |
| *Lsat_1_v5_gn_8_28060* | CCATCGTCTGCCTCTCCGTTATG | CACTCGCTCCTTGACTCTGTTCTG |
| *Lsat_1_v5_gn_8_85820* | GCCAGAATATGCCGTCCAGAGC | CGATGACCTGACCGTTGTAGAAGA |
| *LsTIP41* | GAGAGATTTGCTGGAGGGAAACTA | CCTTTGACTGATGATGTTTGGA |
