# Supplementary material for: Combined analysis of the transcriptome and metabolome provides insights into the fleshy stem expansion mechanism in stem lettuce
Source: Front Plant Sci. 2022 Dec 15;13:1101199. doi: 10.3389/fpls.2022.1101199 (PMC9798005; doi:10.3389/fpls.2022.1101199)
Supplement: Supplementary file 2 [file Table_2.docx]

**Table S2** Transcriptome sequencing data

| **Samples** | **Total Reads** | **Clean reads** | **Clean bases** | **GC Content(%)** | **Q20**  **(%)** | **Q30**  **(%)** | **Mapped Reads** |
| --- | --- | --- | --- | --- | --- | --- | --- |
| S1-1 | 48777528 | 24388764 | 7291823534 | 44.37 | 98.32 | 94.75 | 46,505,721(95.34%) |
| S1-2 | 49660460 | 24830230 | 7417632236 | 44.38 | 98.35 | 94.84 | 47,100,969(94.85%) |
| S1-3 | 50296358 | 25148179 | 7522943082 | 44.25 | 98.36 | 94.84 | 47,970,802(95.38%) |
| S2-1 | 44339034 | 22169517 | 6628580314 | 44.72 | 97.74 | 93.46 | 41,816,880(94.31%) |
| S2-2 | 46998070 | 23499035 | 7023243632 | 44.58 | 98.19 | 94.38 | 44,699,155(95.11%) |
| S2-3 | 42420998 | 21210499 | 6335505014 | 44.52 | 98.13 | 94.25 | 40,209,167(94.79%) |
| S3-1 | 55461586 | 27730793 | 8269977622 | 43.65 | 97.97 | 94.09 | 51,909,782(93.60%) |
| S3-2 | 49711998 | 24855999 | 7412114290 | 44.11 | 98.12 | 94.5 | 46,755,089(94.05%) |
| S3-3 | 49597610 | 24798805 | 7404886918 | 43.98 | 98.02 | 94.25 | 46,738,165(94.23%) |
| S4-1 | 52224704 | 26112352 | 7792148378 | 44.06 | 97.86 | 93.81 | 49,072,950(93.97%) |
| S4-2 | 53479104 | 26739552 | 7980919426 | 44.18 | 97.78 | 93.61 | 50,047,720(93.58%) |
| S4-3 | 53896386 | 26948193 | 8049832122 | 44.41 | 97.77 | 93.56 | 49,955,923(92.69%) |
